# Thermal and Optical Activation Mechanisms of Nanospring-Based Chemiresistors

**DOI:** 10.3390/s120505608

**Published:** 2012-05-02

**Authors:** Vladimir Dobrokhotov, Landon Oakes, Dewayne Sowell, Alexander Larin, Jessica Hall, Alexander Barzilov, Alex Kengne, Pavel Bakharev, Giancarlo Corti, Timothy Cantrell, Tej Prakash, Joseph Williams, Leah Bergman, Jesse Huso, David McIlroy

**Affiliations:** 1 Department of Physics and Astronomy, Western Kentucky University, Bowling Green, KY 42101, USA; E-Mails: landon.oakes@gmail.com (L.O.); dewayne.sowell363@topper.wku.edu (D.S.); alexander.larin375@topper.wku.edu (A.L.); jessica.hall228@topper.wku.edu (J.H.); alexander.barzilov@wku.edu (A.B.); 2 Department of Physics, E&P Bldg, University of Idaho, Moscow, ID 83844, USA; E-Mails: foue3398@vandals.uidaho.edu (A.K.); bakh8413@vandals.uidaho.edu (P.B.); corti@gonano-9.com (G.C.); lbergman@uidaho.edu (L.B.); huso4579@uidaho.edu (J.H.); dmcilroy@uidaho.edu (D.M.); 3 GoNano Technologies, 112 Sweet Ave., Moscow, ID 83843, USA; E-Mails: Cantrell@gonano-9.com (T.C.); tejasvi.prakash@vandals.uidaho.edu (T.P.); 4 ElectroOptics Research Institute and Nanotechnology Center, University of Louisville, Louisville, KY 40292, USA; E-Mail: jpwill09@louisville.edu

**Keywords:** nanosprings, sensor, electronic nose

## Abstract

Chemiresistors (conductometric sensor) were fabricated on the basis of novel nanomaterials—silica nanosprings ALD coated with ZnO. The effects of high temperature and UV illumination on the electronic and gas sensing properties of chemiresistors are reported. For the thermally activated chemiresistors, a discrimination mechanism was developed and an integrated sensor-array for simultaneous real-time resistance scans was built. The integrated sensor response was tested using linear discriminant analysis (LDA). The distinguished electronic signatures of various chemical vapors were obtained at ppm level. It was found that the recovery rate at high temperature drastically increases upon UV illumination. The feasibility study of the activation method by UV illumination at room temperature was conducted.

## 1. Introduction

Functionalized metal oxides are widely used for vapor sensing applications [1–34]. As vapor-sensitive materials they have multiple advantages: high sensitivity, short time of response, and self-refreshability. A productive approach in sensor design is the use of metal oxide-based nanomaterials as the gas sensitive layer. Because of their small size, the internal bulk of the nanomaterials can be very susceptible to changes at their surface due to the fact that the length scales of surface interactions can be comparable to the dimensions of the nanomaterial. For these reasons, nanostructures have been targeted as materials of choice for chemiresistor construction. The sensing mechanism of metal oxide nanostructures is based on the activation of atmospheric oxygen on the surface at high temperatures [1–26]. Consequently, the catalytic reactions of gaseous species with oxygen sites on the surface induce charge transfer from the surface to the bulk, which changes the electrical resistance of the device. Despite the multiple attractive features of metal oxides as gas-sensitive materials, the detection of chemicals with a high-temperature chemiresistor can be challenging and presents a number of difficulties, such as: substantial thermal noise, high energy consumption and large size from the incorporation of additional heating elements, temperature controller, and signal processing elements. Also, keeping the device at high temperature reduces its durability. Approaches taken to address these problems require an alternative to the high temperature mechanism of activation. A few successful attempts were taken to utilize an optical activation mechanism by exposing metal oxide films and nanowires to UV radiation [31–34]. However, a consistent understanding of UV activation, as well as the comparison of UV *vs.* thermal activation, has yet to be discussed.

**Figure 1.**
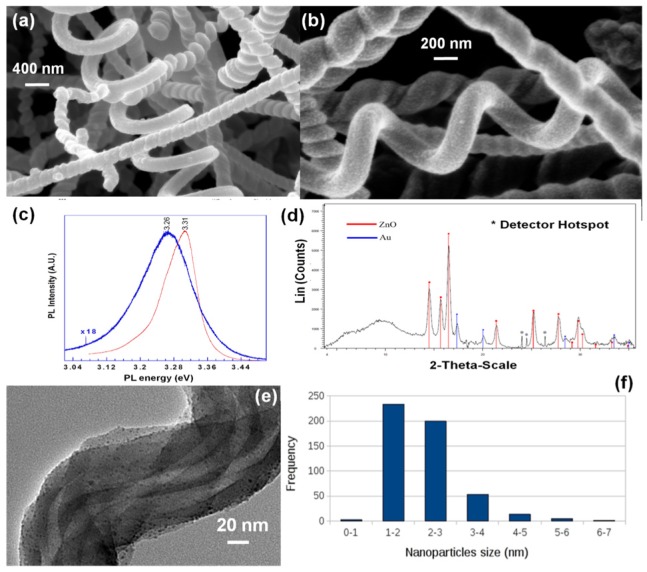
(**a,b**) SEM images of various ZnO coated nanosprings. (**c**) Room temperature photoluminescence spectra ZnO coated silica nanosprings (blue) and a single-crystal ZnO reference (red). (**d**) 2θ-XRD rocking curve for ZnO coated nanosprings. (**e**) A TEM images of silica nanosprings, sans ZnO, coated with Pd nanoparticles. (**f**) The Pd nanoparticle size distribution.

In this paper we present the outcomes of a study on a new type of chemiresistor based on ZnO coated silica nanosprings (Figure 1(a,b)). In this chemiresistor, the mat of insulating nanosprings serves as scaffolding for 10–20 nm nanogranular ZnO coatings distributed over the 400 m^2^/g of accessible surface area of nanosprings. This design incorporates the best gas-sensitive properties of nanostructures with the simplicity of thin films: ultra-high surface-to-volume ratio and controllable thickness and morphology of the sensor material. We discuss the effects of UV and thermal activation on the nanospring-based sensor response and compare the magnitudes and times of response, times of reset and other sensor characteristics with the type of activation. On the basis of our measurements and observations, a consistent model of a UV-activated sensor is presented. Also, we demonstrate how exposure of the thermally activated sensor to UV after each cycle of exposure improves the desorption of chemicals from the surface and drastically decreases the time of reset.

## 2. Material Synthesis and Characterization

Nanospring mats were grown via a vapor-liquid-solid (VLS) mechanism. A detailed description of the process was previously reported by Wang *et al.* [35] and McIlroy *et al.* [36]. Nanospring synthesis was performed in a furnace operated at atmospheric pressure. The process uses a gold layer as a catalyst on a Si substrate, which is then exposed to a proprietary silicon precursor [35–37] as well as a constant O_2_ flow rate concomitant with the silicon precursor. The growth time for an 80 μm thick nanospring-mat is approximately 15 minutes. The nanosprings were coated with ZnO using the ALD technique (Figure 1(a,b)).

ALD utilizes a binary reaction sequence of self-saturating chemical reactions between gaseous precursor molecules and a solid surface to deposit films in a monolayer-by-monolayer fashion [38,39]. We argue that ALD coating of silica nanosprings with ZnO has several advantages over self-assembled nanowire mats. Namely, control over the coating thickness and subsequent nanocrystal size and doping is readily achieved. ALD coating of ZnO was carried out in a tube furnace maintained at 170 °C using diethyl zinc and water as the precursors [39]. Between each pulse of diethyl zinc, or water, the system was purged with N_2_. This ensured that the precursors did not react in the vapor phase and only at the surface of the nanosprings [40]. At low growth temperatures ZnO films produced by ALD retain their favorable properties, enabling construction of hybrid nanoscale organic/ZnO substrates [41,42]. The cycling times are specific to each deposition system, where in this study precursor cycle times were approximately ∼0.25 s or less, and purge and pump times 20–25 s. The thickness of the ZnO layer is controlled by the number of cycles and the purge and precursor pulse widths. Following the above procedure for 200 cycles, a uniformly distributed nanocrystalline ZnO coatings were achieved. Subtle changes in the pump and purge times provides control over the size of the ZnO nanocrystals.

SEM imaging of ZnO/nanosprings shows that the range of average grain sizes is between 3 nm and 100 nm, depending on the ALD conditions. The electronic quality of the ZnO nanocrystals, *i.e.*, the existence of defects, *etc.*, was evaluated by photoluminescence. The photoluminescence (PL) of bare ZnO coated silica nanosprings (ZnO-NS) was analyzed using a CW-Kimmon laser with a wavelength of 325 nm (3.8 eV) together with a JY-Horiba micro-Raman/PL system consisting of a high-resolution T-64000 triple monochromator and a UV microscope capable of focusing to a spot size of ∼800 nm in diameter. Representative room temperature PL spectra of the ZnO coated silica nanosprings (blue) and a single-crystal ZnO reference (red) are displayed in Figure 1(c). The peak of the PL of the ZnO-NS is ∼50 meV lower in energy than that of the single-crystal ZnO. Our previous study on the PL properties of ZnO nanocrystals and nanostructured flexible ZnO thin films found similar results [43,44], where the lower emission was attributed to surface defects. Schirra *et al.* [45] utilized cathodoluminescence, in conjunction with high resolution imaging, to show that this type of PL originates predominantly from structural defects on ZnO surfaces, such as stacking faults. Furthermore, the broad PL line of the ZnO-NS relative to that of the single crystal is also an indicator of the presence of defects. The crystal structure of the ZnO nanocrystals was verified with X-ray diffraction. Displayed in Figure 1(d) is a representative 2θ-XRD rocking curve for ZnO-NS. The primary peaks of ZnO are all present (red bars), as well as those of Au (blue bars), where Au is the catalyst used for nanospring synthesis. The peaks with * are artifacts of hotspots of the x-ray detector. The XRD spectrum is indicative of good quality ZnO.

In addition to ZnO coated nanosprings, metal nanoparticle-decorated ZnO coated nanosprings were also fabricated (Figure 1(e)). Metal-mediated interactions are expected to increase the sensitivity and selectivity to gases and vapors [21]. Metal nanoparticles were synthesized on the ZnO coated nanosprings by wet impregnation [21]. Acetylacetonante (acac)metal-organic salts were dissolved in an organic solvent, such as ethanol or benzene, with a typical base concentration of 1.95 × 10^−2^ M of metal content, where the solvent and final concentration varies depending on the material to be deposited. For example, palladium nanoparticles were obtained with a solution of Pd(II) aceylacetonate (Strem Chemicals). For a 1 cm × 1 cm planar sample of nanosprings 10.4 microliters of the solution was applied to the sample surface and the solvent allowed to evaporate at room temperature. Next, the sample was placed in an oven at 500 °C under a hydrogen-nitrogen flow for 15 minutes, thereby reducing the compound to Pd nanoparticles. Finally, the sample was allowed to cool to room temperature in a nitrogen atmosphere to avoid oxidation. The TEM image in Figure 1(e) verifies that this procedure produces a Pd NP size distribution between 2–5 nm, with an average size of 2.4 ± 1.3 nm (Figure 1(f)).

## 3. Thermal Activation of Chemiresistors

**Figure 2.**
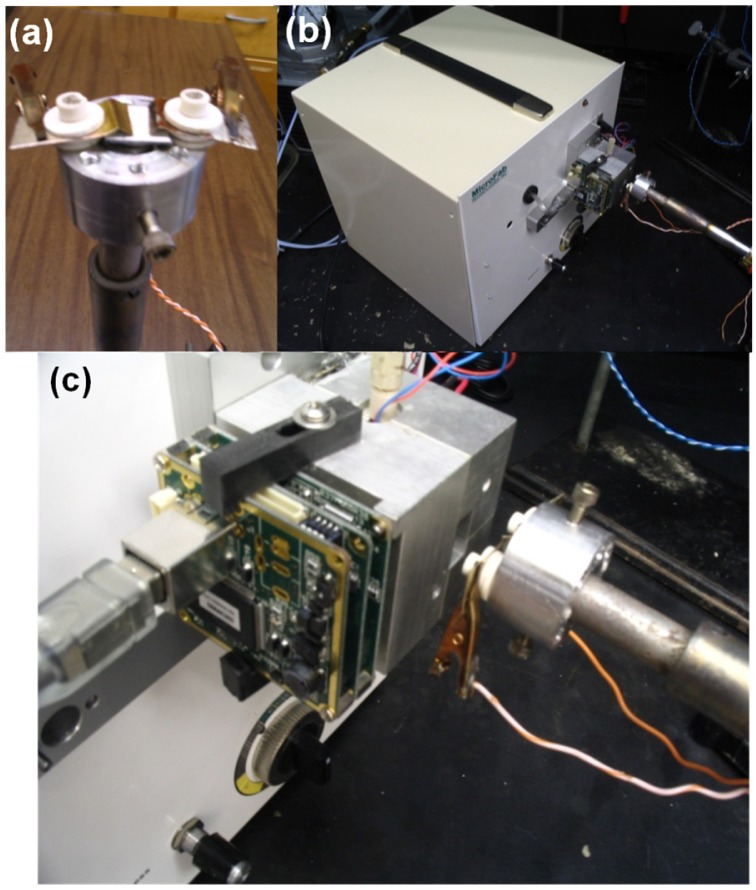
Images of the experimental setup: (**a**) the sensor holder, (**b**) the sensor holder in front of the output of the VaporJet, and (**c**) a close up view of the sensor holder in front of the Vaporjet.

A standard two electrode test geometry was used for measuring the electrical response of the nanospring-based chemiresistors to chemical vapors. The electrodes were annealed and the ohmic nature of ZnO-metal contacts was verified by I-V characterization. The sensor was connected to a thermocouple and placed on a variable temperature platform for temperature control, as shown in the Figure 2(a). The sensor responses were acquired with a Keithley 2400 SourceMeter interfaced to a computer via Labview-operated data acquisition software allowing for real time conductance measurements. A continuous flow of synthetic air (20% O_2_ and 80% N_2_) on the sensor was maintained at all times. The sensor was initially heated to 400 °C in synthetic air at ambient pressure to obtain a steady state resistance. For an average ZnO coating, the resistance upon heating changes from a few kΩ to tens of MΩ. Temperature dependencies of the electronic properties of ZnO-coated nanosprings is significantly different than bulk ZnO. Heating bulk ZnO excites free carriers, producing a drop in resistance. However, for ZnO coated nanosprings, we observed two competing processes: thermal generation of free carriers (decreases the resistance) and trapping of free electrons by ionized oxygen at the surface (increases the resistance). At high temperatures, the depletion by adsorbed oxygen dominates—a necessary condition for high sensitivity. In fact, the ability to adsorb large quantities of oxygen is the most important factor in determining the sensitivity of a chemiresistor, which is based on rapid catalytic reactions.

Once a steady state conductance was achieved, pulses of vapor were introduced in the air flow by the VaporJet calibrator [46], as shown in Figure 2(b,c). To effectively determine the detection limit of the chemiresistor, sublimation of solids is preferable to the evaporation of liquids. The VaporJet's ability to sublimate solids almost instantly allows for extremely short pulse on the order of milliseconds [46]. The performance of the Pd/ZnO nanospring sensors was tested using the VaporJet with the explosives trinitrotoluene (TNT) and triacetone triperoxide (TATP) at an operating temperature of 400 °C [47]. The compounds were introduced into the flow of artificial atmosphere in 0.1 ms pulses. Repeatable peaks of conductance were obtained for both chemicals at ppb levels of concentrations.

**Figure 3.**
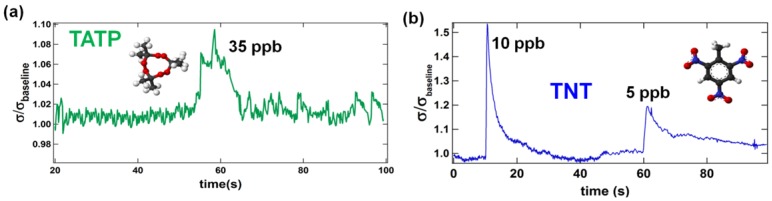
(**a**) The relative change in conductance of a thermally activated Pd/ZnO nanospring sensor upon exposure to TATP at 35 ppb and an exposure pulse of 0.1 ms; (**b**) The relative change in conductance of a thermally activated Pd/ZnO nanospring sensor upon exposure to TNT at 10 ppb and 5 ppb and exposure pulses of 0.1 ms.

Examples of their responses are displayed in Figure 3(a,b). The results demonstrate that the sensors are extremely sensitive to ppb concentrations and short exposure times. A consequence of these characteristics is their quick recovery of 20–40 s, which demonstrates their superiority to previously reported designs [1–34]. Specifically, the best reports on vapor detection of ppb concentrations required several seconds to a few minutes of exposure, *i.e.*, an accumulation of adsorbed vapor onto the sensor's surface was required to achieve a detectable level of response. Examples of their responses are displayed in Figure 3(a,b). The results demonstrate that the sensors are extremely sensitive to ppb concentrations and short exposure times. A consequence of these characteristics is their quick recovery of 20–40 s, which demonstrates their superiority to previously reported designs [1–34]. Specifically, the best reports on vapor detection of ppb concentrations required several seconds to a few minutes of exposure, *i.e.*, an accumulation of adsorbed vapor onto the sensor's surface was required to achieve a detectable level of response. The nearly instantaneous and reversible response of nanospring based sensors to ultra-small vapor concentrations bodes well for their realization as field deployed sensors of explosive. The difference in the detection limit of TNT and TATP (Figure 3(a,b)) is currently attributed to the specific thermodynamic characteristics of the respective catalytic reactions. Contrary to most conventional nitrogen-containing explosives (TNT, PETN, RDX) which transfer much of their energy into heat through rapid catalytic oxidation, peroxide-based explosives, such as TATP, undergo entropic catalytic oxidation resulting in an almost instantaneous decomposition of every solid state TATP molecule into four gas-phase molecules—one ozone and three acetone molecules [48].

The nearly instantaneous and reversible response of nanospring based sensors to ultra-small vapor concentrations bodes well for their realization as field deployed sensors of explosive. The difference in the detection limit of TNT and TATP (Figure 3(a,b)) is currently attributed to the specific thermodynamic characteristics of the respective catalytic reactions. Contrary to most conventional nitrogen-containing explosives (TNT, PETN, RDX) which transfer much of their energy into heat through rapid catalytic oxidation, peroxide-based explosives, such as TATP, undergo entropic catalytic oxidation resulting in an almost instantaneous decomposition of every solid state TATP molecule into four gas-phase molecules—one ozone and three acetone molecules [48].

## 4. Integrated Sensor Design and Response

**Figure 4.**
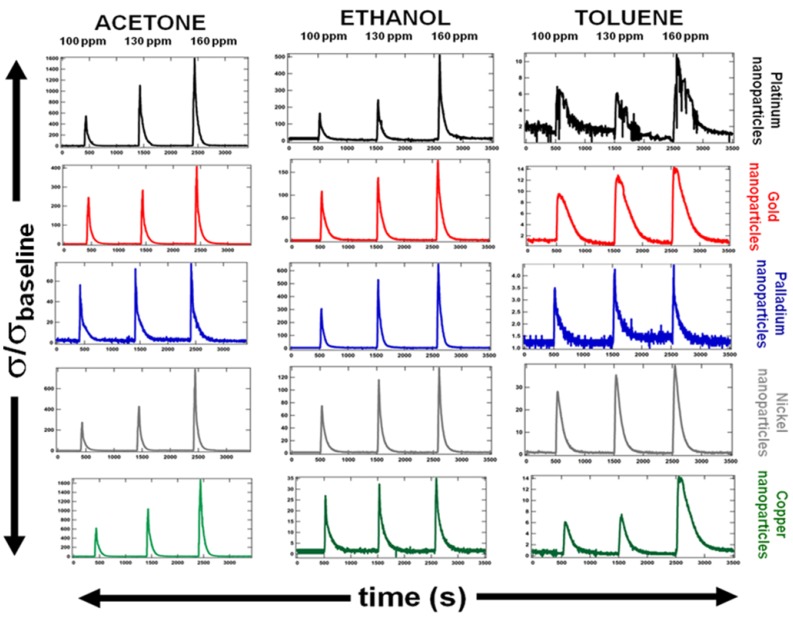
Integrated matrix of responses to tree types of chemical vapors. Each column corresponds to a particular vapor: acetone, ethanol, or toluene. Each row corresponds to a particular type of sensor, which is determined by the type of metal nanoparticles in the coating. Consequent peaks correspond to tree different partial vapor pressures (100, 130, and 160 ppm). The sensor response is measured as conductance normalized with respect to a baseline signal level, when no vapor is present.

In order to conduct a feasibility study of an artificial olfactory system based on functionalized nanosprings, the simultaneous response of several sensors, coated with the following metal nanoparticles: Pt, Pd, Au, Ni, and Cu, was investigated. To test the electrical response of chemiresistors to chemical vapors, five samples (#1—ZnO/Pt nanoparticles coated, #2—ZnO/Au nanoparticles coated, #3—ZnO/Pd nanoparticles coated, #4—ZnO/Ni nanoparticles coated, and #5—ZnO/Cu nanoparticles coated) were assembled into an integrated array and placed into a tubular flow furnace at 400 °C. Each sensor in the array was connected to Labview-operated data acquisition software through a National Instruments NIcompactDAQ9178 chassis in conjunction with NI9264 voltage source and the NI9203 ammeter, for simultaneous real-time resistance scans. A continuous flow of dry air into the furnace was maintained at all times. Basic degradation products of high-explosives were chosen as analytes for this study. Sequential vapor pulses were introduced into the flow-tube via a VaporJet calibrator. Figure 4 summarizes the synchronized matrix of responses of the five sensors to acetone, ethanol and toluene. Each row corresponds to a particular type of sensor, determined by the type of metal nanoparticle coating, while each column corresponds to a particular vapor. Sequential peaks in the graphs correspond to vapor pulses with concentrations of 100 ppm, 130 ppm, and 160 ppm, respectively. The sensor response is measured as conductance normalized to their respective baseline signals in the absence of vapor. Although the chemiresistors are cross-sensitive to multiple gases, the vertical scales drastically differ from graph to graph. For instance, the response of Pd coated ZnO to 160 ppm of acetone is ∼1,500 units, to 160 ppm of ethanol is ∼500 units, and to 160 ppm of toluene is ∼12 units. The relationship between individual sensor responses in the integrated signal appears to be significantly different.

**Figure 5.**
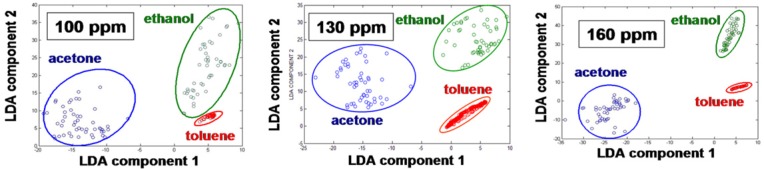
Separation of classes of chemicals in two-dimensional slices of multidimensional hyperspace using LDA technique at three different vapor pressures.

The Linear Discriminant Analysis (LDA) method was used for separation and recognition of vapors. LDA is a commonly used technique for pattern analysis [1,49]. It has been widely used and proven successful in many applications. Figure 5 demonstrates the LDA analysis of sensor responses shown in the Figure 4. Ten second intervals of the sensor response were used to create transient signals for each of the vapors detected. From these transient signals, common features were extracted to create a feature vector for each sensor. These features include: the signal amplitude, total area beneath the adsorption curve, total area beneath the response curve, and time for the signal to reach its amplitude value [1,49]. LDA is used to find a projection which minimizes within class variance while maximizing the distance between class means. This was accomplished by finding a projection which maximizes Fisher's criteria. Responses were then projected onto the Fisher basis vectors to obtain discrimination. The principal components were calculated for three different vapor pressures. As expected, a drop in vapor pressure makes the chemicals less distinguishable (Figure 5). The primary mechanism for increasing the discrimination power of an integrated sensor array is the number of sensors containing different nanoparticles.

To ensure the consistency and reliability of the integrated sensor response, a range of relatively high vapor pressures (between 100 and 200 ppm) and well-known chemical vapors was considered. This selection of vapors was used to conduct a feasibility study of selectivity and specificity of the functionalized nanosprings and to obtain electronic signatures of vapors at different vapor pressures. This selection did not diminish the role of sensitivity and a detection limit of the sensor was obtained along with evidence regarding the ability to recognize more complex chemical vapors. Nanospring-based sensors demonstrate promising capabilities to detect complex vapors such as TNT and TATP at ppb concentrations (Figure 3). However, reliable pattern recognition at this level of signal requires additional pre-processing and noise reduction techniques, which will be left for the future publications.

## 5. UV Reset Mechanism of Thermally Activated of Chemiresistors

Besides sensitivity and selectivity, the level of noise and baseline stability are important factors in determining overall sensor performance. Baseline stability refers to the ability of a sensor to refresh completely after each cycle of exposure. Due to the fast evaporation of the catalytic reaction products from the ZnO surface, nanospring-based sensors are self-refreshing and, in general, do not need a reset mechanism. For gases like CO, where the oxidation to CO_2_ is a simple one-step catalytic reaction, the recovery rate is faster compared with vapors and the recovery curve is more smooth [3–26]. Upon exposure to simple vapors such as toluene or ethanol the recovery time is longer; however, the recovery persists for a few seconds with complete baseline restoration [3–26]. For complex organic analytes (TNT, TATP, RDX, HMX) with several intermediate oxidation stages, it is highly desirable to reduce the recovery time in order to achieve proper efficiency and accuracy. For example, TNT (C_7_H_5_N_3_O_6_) is able to undergo full catalytic oxidation with products of carbon dioxide, water and nitrogen gas. However, TNT has a negative oxygen balance, which implies that the oxygen content of the molecule is insufficient compared to the amount of oxygen needed to form all of these products. Thus, as with many explosives, TNT's oxidation is more efficient in the presence of an external oxidant, implying that the oxygen-rich high-temperature surface of ZnO comprises an environment favorable for the following catalytic reaction:

(1) $$2C_{7}H_{5}N_{3}O_{6}+21O^{-}\to 14CO_{2}+5H_{2}O+3N_{2}+21e^{-}$$

**Figure 6.**
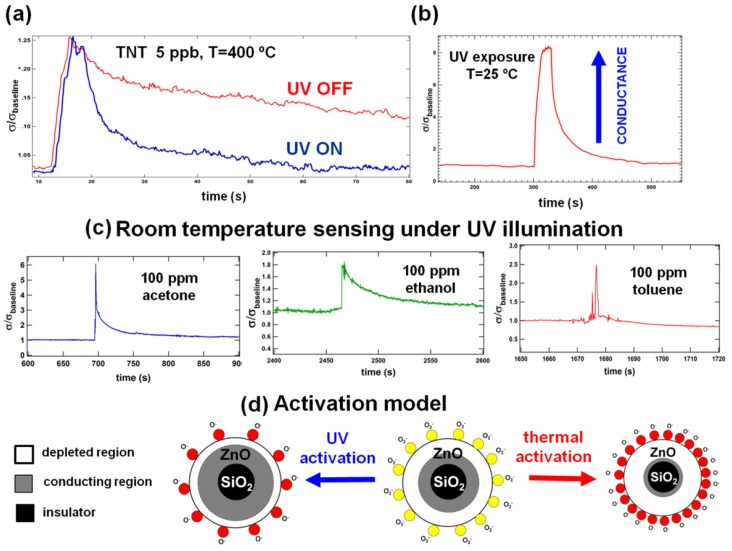
(**a**) The relative change in conductance of a thermally activated Pd/ZnO nanospring sensor upon exposure to TNT at 5 ppb and exposure pulses of 0.1 ms. The upper desorption curve corresponds to a self-reset, and the lower one corresponds to a reset upon the UV exposure; (**b**) The relative change in conductance of Pd/ZnO nanospring sensor upon a 20 s UV exposure in the ambient atmosphere at room temperature; (**c**) The relative change in conductance of a UV activated Pd/ZnO nanospring sensor upon exposure to vapor-phase analytes at 100 ppm and an exposure pulse of 1 s; (**d**) A cross-section view of a nanowire showing the size of the conducting region (grey) with no activation at room temperature (center), upon the thermal activation (right), and upon the UV-activation (left).

During this reaction, some of the TNT molecules experience only partial oxidation upon reaching the ZnO surface. The intermediate reaction products (dinitrotoluene, toluene *etc.*) remain attached to surface defects and then oxidize at a slower rate to the final oxidation stage gaseous products. These products evaporate from the surface, causing re-adsorption of atmospheric oxygen and thus baseline restoration. The average thermal energy of electrons in bulk ZnO may not be high enough to ionize atmospheric oxygen into *O*^–^ groups on the surface. Only a small fraction of high-energy electrons are involved in the oxygen ionization, creating a slow recovery rate. These processes together cause the step-like behavior observed in the desorption portion of the vapor-sensing characteristics (Figure 6(a)) which is often erroneously attributed to thermal noise despite the amplitude of these steps being sufficiently higher than the noise. This step-like behavior is not observed in the sensing characteristics of simple gases, where the full catalytic oxidation occurs in a single-step. For instance, carbon monoxide oxidizes to carbon dioxide via the following reaction:

(2) $$\text{CO}+O^{-}\to CO_{2}+e^{-}$$

To increase the sensor recovery rate after a TNT pulse, the nanospring-mat was exposed to broad-spectrum UV radiation. ZnO is well known for its high optical activity [31–34]; therefore, when this wide band gap material (band gap 3.4 eV) is illuminated under UV light with energy equal or greater than 3.4 eV, a large number of high-energy electron-hole pairs (excitons) is generated. In addition, the products of partial TNT oxidation can be cracked by UV light and oxidized by the activated oxygen on the surface. Once the gaseous products of full oxidation (CO_2_, H_2_O, and N_2_) desorb from ZnO, the high energy electrons generated by UV light ionize the atmospheric oxygen on the surface and restore *O*^−^ coverage. The experimental observation of this effect is shown in the Figure 6(a), where the recovery rate at high temperature drastically increases upon the UV illumination.

## 6. Room Temperature UV Activation of Chemiresistors

At room temperature, the exposure of nanosprings to UV light in the ambient air causes an increase in conductance by a factor of eight (Figure 6(b)). This jump in conductance is attributed to the enhanced carrier density in the nanospring and the reduced depletion width. Once the electron-hole pairs are generated by the UV light, the holes migrate to the surface and discharge the adsorbed oxygen ions. This causes the depletion layer width and the intergranule barrier height to decrease, resulting in the desorption of surface oxygen. Over time, the unpaired electrons accumulate until the desorption and adsorption of oxygen reaches an equilibrium state. Hence, UV excitation leads to an enhanced carrier density in the nanospring and a narrower depletion width, resulting in a gradual increase in current until saturation.

The UV-activated room-temperature gas sensing characteristics for the ZnO/Pd coated sensor upon exposure to a 1 sec pulse of vapor phase analytes at 100 ppm are shown in the Figure 6(c). The UV-activated sensor responds to a vapor pulse and returns to the steady state faster than if it were thermally activated and does so with a significantly lower level of noise. However, the response level of the UV-activated sensor is more than ten times lower than the response of a thermally activated one. The measurable response to ppb levels of TNT and TATP at room temperature was not observed. Although the sensing mechanism for thermally-activated ZnO and UV-activated ZnO are the same, the steady state conditions for them are qualitatively different. For the thermally-activated sensor, a significant increase in resistance in the steady state due to the depletion of ZnO by the adsorbed oxygen is an indicator of high sensitivity. In contrast, for a UV-activated sensor in the steady state under constant UV illumination the resistance drops, indicating that the amount of adsorbed oxygen is less than for the non-UV room temperature conditions. Although it seems like a paradox, the mechanism of this effect is hidden in the nature of the adsorbed oxygen species. The mechanism of UV-activation of a ZnO sensor is shown in the Figure 6(d). Under non-UV conditions ionized oxygen chemisorbs on the surface in its molecular form, *O*_2_^−^. In this form it is less interactive: analogously, the sensitivity of high-temperature sensors is very low below 150 °C, when the molecular form prevails. When UV exposed ZnO reaches the steady-state adsorption-desorption equilibrium, the amount of adsorbed oxygen decreases compared to non-UV conditions. However, the presence of high energy excitons leads to the formation of the atomic form of adsorbed oxygen, *O*^−^, which is substantially more chemically interactive and creates favorable conditions for catalytic reactions.

## 7. Conclusions

We have developed a method for constructing high surface area chemiresistors using silica nanosprings as scaffolding. The chemiresistors were fabricated by coating the silica nanosprings by atomic layer deposition with ZnO, or ZnO followed by decoration with Pd nanoparticles. Thermal and optical methods of sensor activation were developed and compared. At 400 °C, well-defined spikes in conductance upon exposure to explosive vapors (TNT, TATP) were obtained for 0.1 ms exposure times at ppb levels. It was found that exposure of the thermally activated sensor to UV light after each cycle of exposure improves the desorption of chemicals from the surface and drastically decreases the time of reset. Room temperature UV-activation of solid state sensors has multiple attractive features, such as: sufficient noise reduction, stable baseline, short time of response and recovery, long durability and low energy consumption. However, the detection limit is sufficiently higher for the thermally activated sensors. Therefore, until the UV mechanism is fully developed, thermal excitation remains the primary mechanism for activation in oxide-based chemical sensors.
